# ADAM9 promotes lung cancer progression through vascular remodeling by VEGFA, ANGPT2, and PLAT

**DOI:** 10.1038/s41598-017-15159-1

**Published:** 2017-11-08

**Authors:** Chen-Yuan Lin, Chia-Fong Cho, Shih-Ting Bai, Jing-Pei Liu, Ting-Ting Kuo, Li-Ju Wang, Yu-Sen Lin, Ching-Chan Lin, Liang-Chuan Lai, Tzu-Pin Lu, Chih-Ying Hsieh, Chin-Nan Chu, Da-Chuan Cheng, Yuh-Pyng Sher

**Affiliations:** 1 Graduate Institute of Clinical Medical Science, China Medical University, Taichung, 404 Taiwan; 2 Graduate Institute of Biomedical Sciences, China Medical University, Taichung, 404 Taiwan; 3 Department of Biomedical Imaging and Radiological Science, China Medical University, Taichung, 404 Taiwan; 4 Center for Molecular Medicine, China Medical University Hospital, Taichung, 404 Taiwan; 5 Division of Hematology and Oncology, China Medical University Hospital, Taichung, 404 Taiwan; 6 Division of Thoracic Surgery, China Medical University Hospital, Taichung, 404 Taiwan; 7 Department of Radiation Oncology, China Medical University Hospital, Taichung, 404 Taiwan; 8 Graduate Institute of Physiology, National Taiwan University, Taipei, 106 Taiwan; 9 Department of Public Health, National Taiwan University, Taipei, 106 Taiwan

## Abstract

Lung cancer has a very high prevalence of brain metastasis, which results in a poor clinical outcome. Up-regulation of a disintegrin and metalloproteinase 9 (ADAM9) in lung cancer cells is correlated with metastasis to the brain. However, the molecular mechanism underlying this correlation remains to be elucidated. Since angiogenesis is an essential step for brain metastasis, microarray experiments were used to explore ADAM9-regulated genes that function in vascular remodeling. The results showed that the expression levels of vascular endothelial growth factor A (VEGFA), angiopoietin-2 (ANGPT2), and tissue plasminogen activator (PLAT) were suppressed in ADAM9-silenced cells, which in turn leads to decreases in angiogenesis, vascular remodeling, and tumor growth *in vivo*. Furthermore, simultaneous high expression of ADAM9 and VEGFA or of ADAM9 and ANGPT2 was correlated with poor prognosis in a clinical dataset. These findings suggest that ADAM9 promotes tumorigenesis through vascular remodeling, particularly by increasing the function of VEGFA, ANGPT2, and PLAT.

## Introduction

Lung cancer can progress quickly, with multiple metastases in its later stages. The 5-year relative survival rate of metastatic lung cancer is only 2%^1^. The incidence of brain metastases is particularly high in patients with lung tumors: 10–25% of lung cancer patients have brain metastases at diagnosis and another 50% develop brain metastases during the remaining course of the disease^2^. Currently, mortality caused by distant metastases, especially brain metastases, remains the major dilemma in lung cancer. Metastasis to the brain from the primary lung tumor requires multiple molecular mechanisms for cancer cells, including survival while in circulation, vascular remodeling, angiogenesis, and immune evasion. Thus, exploring the mechanisms of lung cancer metastasis to the brain may help to identify and develop potential strategies for lung cancer treatment.

Vascular remodeling and early angiogenesis are essential for successful brain metastasis formation^3^. Vascular remodeling in cancer via generation of new vessels (angiogenesis) is required for tumor growth and to provide a route of transport for metastatic tumor cells^4^. The altered vascular network relies on activation of endothelial cells by pro-angiogenic molecules and remodeling of cell-cell junctions which are including vascular endothelial cadherin (VE-cadherin), zonula occludens-1 (ZO-1) and others, and the basement membrane^5^. For example, highly expressed vascular endothelial growth factor (VEGF) in cancers plays important role in angiogenesis. Angiopoietins 1 and 2 (ANGPT1 and ANGPT2) bind endothelial Tie2, a tyrosine kinase receptor, to regulate vascular homeostasis^5^. ANGPT1 supports endothelial stabilization via Tie2 activation, but ANGPT2 antagonizes ANGPT1 activity and thereby causes vascular destabilization and permeability^6^. Moreover, tumor endothelial cells express elevated ANGPT2 levels, and this correlates with tumor angiogenesis and poor prognosis in many cancers. Increased expression of VEGFA and ANGPT2 has been reported in human brain endothelial cells with vascular remodeling and blood-brain-barrier (BBB) dysfunction^7,8^. Therefore, VEGF and ANGPT2 are considered as attractive therapeutic targets for anti-angiogenic therapy.

Accumulating evidence has demonstrated that overexpression of a disintegrin and metalloproteinase 9 (ADAM9) promotes cancer cell metastasis^9,10^. ADAM9 is a member of the ADAM families, which are type I transmembrane proteins containing a metalloproteinase domain responsible for shedding and releasing cell surface proteins, including growth factors, cell adhesion molecules, cytokines, or receptors. This biological function of ADAM9 is increased in cancer cells and is linked to promotion of tumor progression^11,12^. Ectopic overexpression of ADAM9 in lung cancer cells results in cancer cells metastasizing to the brain in animal models^13^. From our previous studies, we know that ADAM9 allows lung cancer cells in an anchorage-free state to circumvent anoikis by (i) up-regulating the activity of the pro-migratory CUB domain-containing protein-1 (CDCP1) via cleavage by tissue plasminogen activator (tPA, PLAT) and (ii) reciprocally decreasing expression of plasminogen activator inhibitor 1 (PAI-1, SERPINE1)^14^. Furthermore, ADAM9 can down-regulate microRNA-218, relaxing the suppression of its target gene *CDCP1*, resulting in high levels of CDCP1 and thereby promoting metastasis^15^. Overexpression of ADAM9 increases the shedding of several membrane proteins from endothelial cells to enhance pathological neovascularization, including ephrin type-B receptor 4, Tie-2, and VE-cadherin^16^. Despite the well-known shedding function of ADAM9 in neovascularization, whether ADAM9 has other functions in vascular remodeling remains unknown. In this study, we demonstrate that VEGFA, ANGPT2, and PLAT are up-regulated in ADAM9-expressing lung cancer cells. We show that ADAM9 promotes vascular remodeling in lung cancer cells by increasing the expression of VEGFA, ANGPT2, and PLAT. Lastly, we show that simultaneous high expression of ADAM9 and VEGFA or of ADAM9 and ANGPT2 is correlated with poor prognosis in a clinical dataset. These results suggest a mechanism for how high levels of ADAM9 might promote brain metastases of lung cancer.

## Results

### ADAM9 up-regulates vascular remodeling-associated genes for neovascular formation

**Figure 1:**
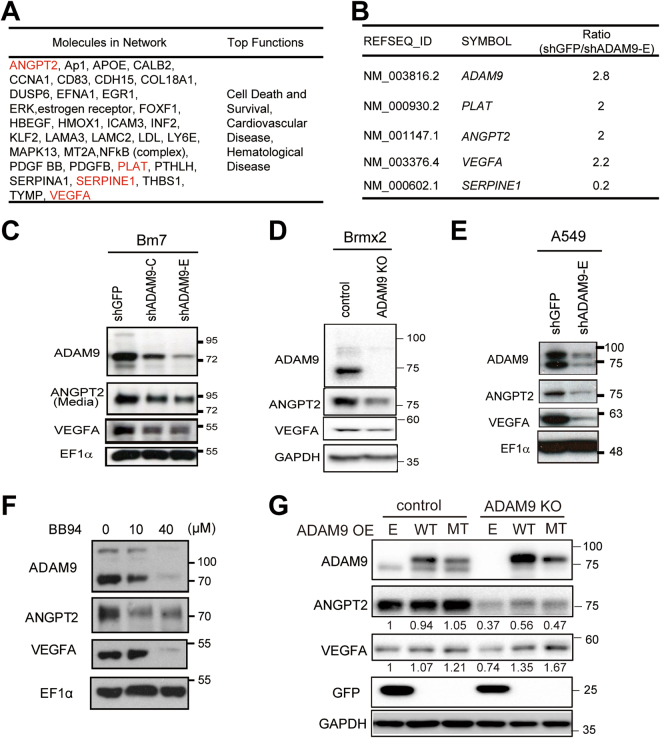
ADAM9-regulated genes with vascular remodeling functions. (**A**) Ingenuity Pathway Analysis, by comparing gene expression in control versus ADAM9 knockdown cells, showed that the gene network functioned in cell death and survival, cardiovascular disease, and hematological disease. (**B**) Ratio of differential gene expression in microarray analysis (control shGFP Bm7 cells/ ADAM9 knockdown Bm7 cells). (**C**–**E**) Western blot of ADAM9, ANGPT2, and VEGFA in control and ADAM9 knockdown cells from Bm7 lung cancer cells (**C**), Brmx2 lung cancer cells (**D**), and A549 lung cancer cells (**E**). (**F**) Western blot of ADAM9, VEGFA, and ANGPT2 in Bm7 cells treated with BB94 (a broad spectrum of metalloproteinase inhibitor). (**G**) Western blot of ADAM9, VEGFA, and ANGPT2 in control and ADAM9 knockout Brmx2 cells transiently transfected with plasmids of empty control (**E**), ADAM9 wild-type (WT), or ADAM9 mutant (MT) (E348A). EF1α or GAPDH served as the loading control in (**C**–**G**). Some cropped blots in (**C**–**G**) were displayed and the full-length blots were shown in Supplementary Figure 1.

**Figure 2:**
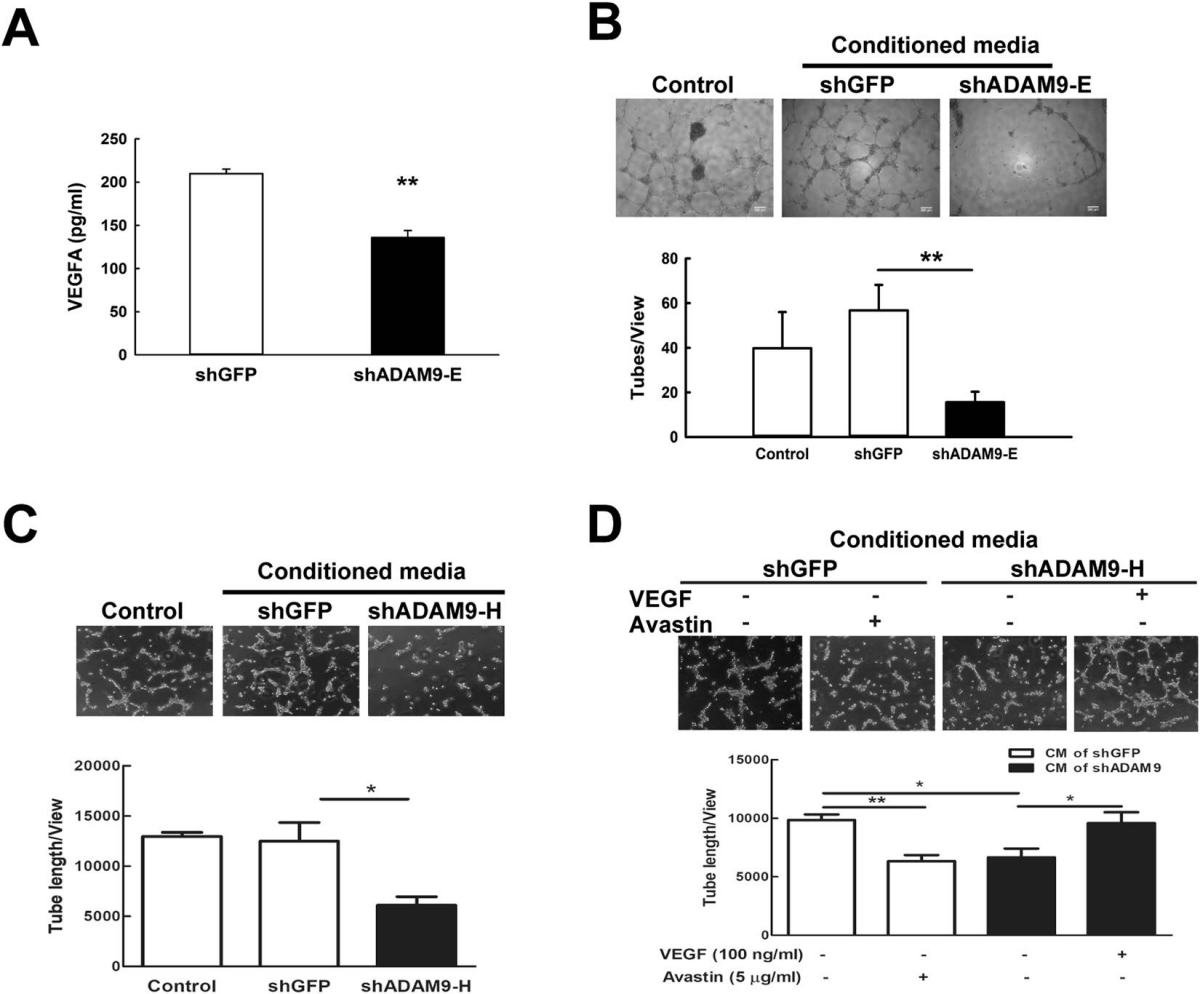
ADAM9 promotes angiogenesis through VEGFA. (**A**) Concentrations of VEGFA were measured by ELISA in conditioned media of control and ADAM9 knockdown Bm7 cells. (**B**,**C**) Tube formation assays of HUVECs were performed by treatment with growth media alone (as control) or the conditioned media of indicated cells. Conditioned media of shADAM9-E Bm7 cells (B) and shADAM9-H Bm7 cells (**C**) were used for tube formation assays. (**D**) Tube formation assays were performed by treating HUVECs with the conditioned media of Bm7-shGFP cells in the presence or absence of 5 μg/ml Avastin pre-incubation (white bars), or with the conditioned media of Bm7-shADAM9 cells in the presence or absence of 100 ng/ml VEGF (black bars). Top, photographs of representative experiments for control, GFP knockdown, and ADAM9 knockdown; bottom, quantitative data for tube formation. Bars represent the mean ± SD of three replicate samples from one representative experiment. *P < 0.05, **P < 0.01.

To determine which genes regulated by ADAM9 in lung cancer cells are involved in vascular remodeling, we performed microarray analysis to identify differentially expressed genes in control and ADAM9 knockdown cancer cells, and then explored pathway analysis to seek genes involved in vascular remodeling. We found that *ANGPT2*, *PLAT* (tPA), *SERPINE1* (PAI-1), and *VEGFA*, which are involved in cardiovascular disease, were influenced in ADAM9 knockdown cells (Fig. 1A). All of these genes had a ≥ 2-fold change in transcription levels (Fig. 1B), except SERPINE1, which had an expression ratio of 0.2 in control cells versus ADAM9 knockdown cells (Fig. 1B). Since we demonstrated that ADAM9 regulates PLAT and SERPINE1 expression in our previous study^14^, here we focused on ANGPT2 and VEGFA. Consistent with RNA expression, protein levels of ANGPT2 and VEGFA were suppressed in ADAM9 knockdown cells compared with the control cells (Fig. 1C). Similar results were also observed in Brmx2 cells (Fig. 1D) and A549 cells (Fig. 1E). To study whether the expression of ANGPT2 and VEGFA was associated with the enzyme activity of ADAM9, BB94, a broad metalloproteinase inhibitor, was added to lung cancer Bm7 cells to check the protein expression. We found that expression of both ANGPT2 and VEGFA was reduced after BB94 treatment in a dose-dependent manner, though the decrease of ANGPT2 protein levels was more obvious than that of VEGFA at 10 μM BB94 (Fig. 1F). Since the expression of ADAM9 was decreased in the presence of BB94, we suggest that ANGPT2 and VEGFA are likely regulated in response to ADAM9 protein levels rather than activity levels, consistent with the result in ADAM9 knockdown cells. Furthermore, to examine the role of metalloprotease of ADAM9 in regulation of ANGPT2 and VEGFA, we constructed the ADAM9 mutant carrying a modification of the conserved glutamic acid in the catalytic domain to alanine (E348A), which can influence the shedding function^17^. The results showed that transient transfection of wild type (WT) or activity mutant (MT) ADAM9 increased ANGPT2 and VEGFA expression in ADMA9 knockout (KO) cells, but that remained in similar levels in control cells (Fig. 1G). It suggests that the protease activity of ADAM9 is not required for regulation of ANGPT2 and VEGFA. Next, to determine the function of ADAM9 in vascular remodeling, we measured the level of secreted VEGFA in conditioned media of cancer cells by ELISA and found lower concentrations of VEGFA in ADAM9 knockdown cell media compared to that in control (shGFP) cell media (Fig. 2A). In addition, HUVECs treated with conditioned media from ADAM9 knockdown cells showed less tube formation than HUVECs treated with media from control (shGFP) cells (Fig. 2B,C). Moreover, the tube formation ability was decreased when conditioned media of shGFP cells was pre-incubated with Avastin, a humanized anti-VEGF monoclonal antibody for neutralizing VEGFA proteins (Fig. 2D). In contrast, increased tube formation was detected in HUVECs treated with media of shADAM9 cells with recombinant VEGFA, indicating VEGF addition can rescue the low tube formation ability of ADAM9 knockdown cells to comparable level in control shGFP cells (Fig. 2D). Taken together, these results suggest that ADAM9 increases the expression of ANGPT2 and VEGFA for neovascular formation.

### ADAM9 contributes to vascular remodeling via ANGPT2

**Figure 3:**
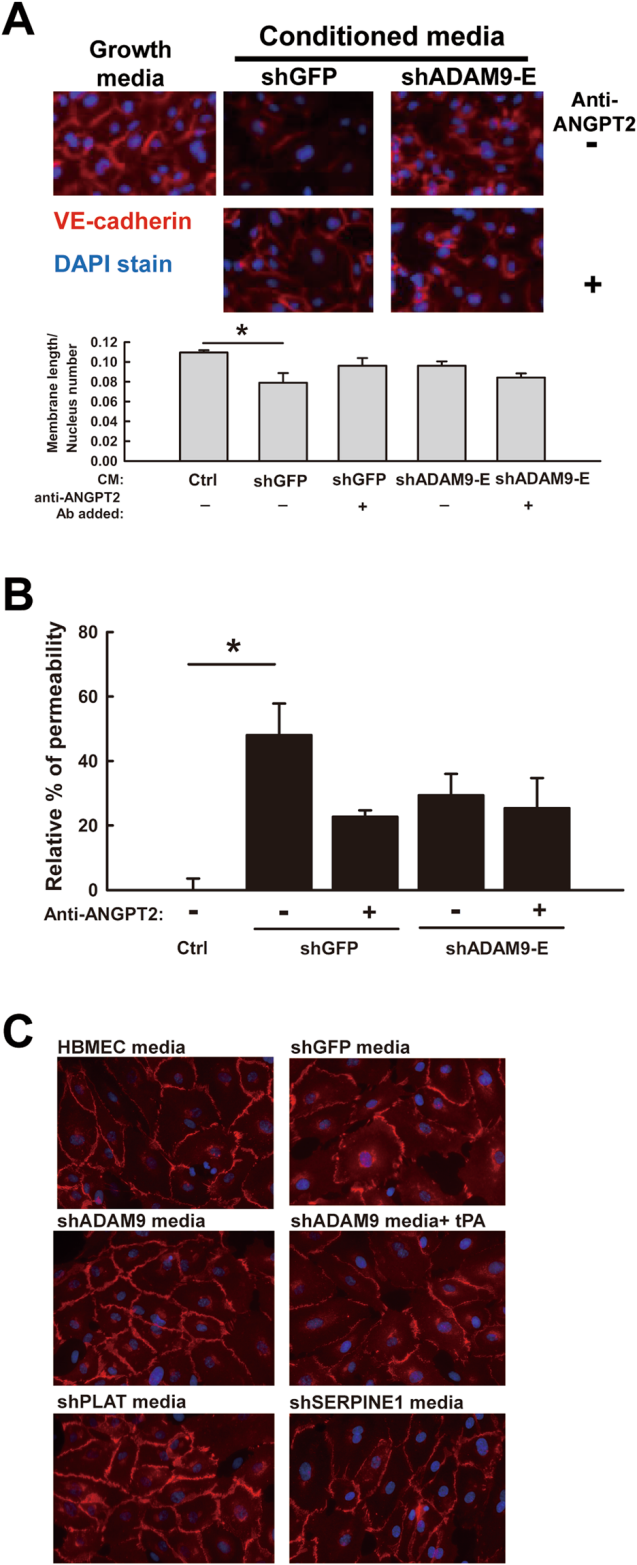
ADAM9 disrupts the integrity of brain endothelial cells through ANGPT2 and PLAT. (**A**) VE-cadherin staining of HBMECs treated with concentrated media from the indicated cancer cells. After incubation, cells were stained with anti-VE-cadherin antibody (red) and DAPI (blue). For quantitation, the membrane length stained by anti-VE-cadherin antibody in HBMECs was calculated and normalized to HBMEC nuclear numbers. Anti-ANGPT2 antibody was pre-incubated with concentrated conditioned media before treatment of HBMECs. (**B**) Permeability of HBMECs treated with concentrated conditioned media from the indicated cancer cells. Anti-ANGPT2 antibody was pre-incubated with concentrated conditioned medium before treatment of HBMECs. ^*^ *P* < 0.05. (**C**) VE-cadherin staining of HBMECs treated with the indicated conditioned media or with recombinant tPA protein reveals the integrity of HBMECs.

To evaluate whether ADAM9-regulated ANGPT2 secretion from brain-metastatic lung cancer cells affects leakage of brain endothelial cells, conditioned media of control and ADAM9 knockdown cells was used to treat HBMECs, followed by detection of VE-cadherin, which is responsible for maintaining the restrictive barrier between endothelial cells^18^. The images showed that HBMECs grew well in normal growth media (Ctrl), with tight and clear surface barriers between cell junctions, but that the cell density of HBMECs decreased in the conditioned media from shGFP cells (Fig. 3A). Moreover, the integrity of the VE-cadherin stained cell surface was lost, but was partially restored by the addition of ANGPT2 antibody in control (shGFP) cell media (Fig. 3A). In contrast, the integrity of the cell surface of HBMECs cultured with ADAM9 knockdown (shADAM9) cell media showed no change, either with or without ANGPT2 antibody (Fig. 3A). Furthermore, we performed a permeability assay to calculate the amount of FITC-albumin that passes through the HBMECs, as an indicator of the loss of cell-cell integrity. The degree of permeability of HBMECs treated with conditioned media from the shGFP cells was significantly higher compared to control cells (media alone), in which HBMEC integrity was maintained (Fig. 3B). The addition of neutralizing anti-ANGPT2 antibody partially restored the permeability (Fig. 3B). Meanwhile, HBMECs treated with conditioned medium of ADAM9 knockdown cells showed a lower degree of permeability compared to shGFP cell-conditioned medium, and anti-ANGPT2 had no effect on the permeability (Fig. 3B). Furthermore, to determine whether ADAM9-mediated vascular remodeling-associated genes influence HBMEC integrity, we compared the HBMEC integrity with VE-cadherin staining in cells treated with the conditioned media from cells with different gene knockdown (shGFP or shADAM9). There was weaker VE-cadherin staining in cells grown in shGFP-conditioned media compared to that with shADAM9–conditioned media. However, addition of recombinant human tPA (PLAT) into shADAM9-conditioned media reduced the tightness of HBMECs, as indicated by the lower VE-cadherin staining (Fig. 3C). In contrast, the integrity of HBMECs treated with conditioned media from PLAT knockdown cells (shPLAT) remained intact, a phenomenon that was reversed in cells treated with conditioned media from plasminogen activator inhibitor 1 (PAI-1, SERPINE1) knockdown cells (Fig. 3C). We have demonstrated that knockdown of SERPINE1 can increase the activity of tPA in lung cancer cells^14^. These results indicate that ADAM9-abundant medium or plasminogen activator-based pathway served as a causative contributor to endothelial cell breakdown and suggest that ANGPT2 and PLAT regulated by ADAM9 from brain-metastatic lung cancer cells disrupts HBMEC integrity. Thus, ADAM9 may enhance vascular remodeling through up-regulating ANGPT2 and PLAT.

### ADAM9 knockout in lung cancer cells reduces tumor progression *in vivo*

**Figure 4:**
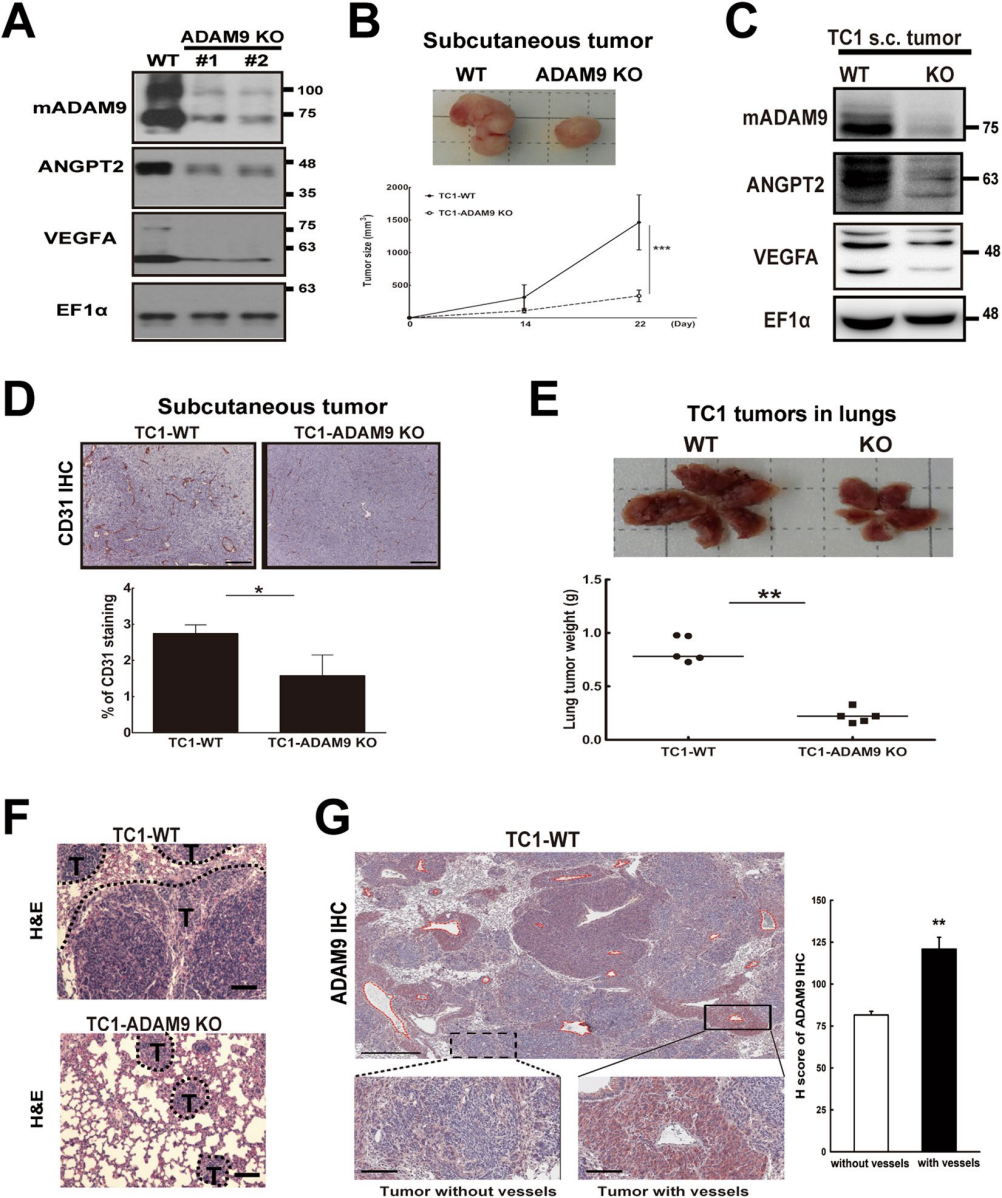
ADAM9 knockout significantly inhibits tumor growth and metastasis *in vivo*. (**A**) Western blot of ADAM9 knockout in mouse lung cancer TC1 cells. WT, wild-type; #1 and #2 represent two independent stable clones with ADAM9 knockout. (**B**) Wild-type (*n* = 7) or ADAM9 knockout (*n* = 7) TC1 cells were injected subcutaneously into C57BL/6 mice. The photograph (*top*) shows subcutaneous tumors removed from each group after sacrifice; the graph (*bottom*) shows the quantification of tumor volume at the indicated days after inoculation. (**C**) Western blot of ADAM9, ANGPT2, and VEGFA from subcutaneous (s.c.) tumors of each group. (**D**) Tumor tissues were processed for IHC staining of CD31. The images were magnified at 100x (*top*, scale bar 200 μm). CD31 staining was quantified as the percentage of positively stained density (*bottom*). (**E**) WT (*n* = 5) and ADAM9 KO (*n* = 5) TC1 cells were injected intravenously via the tail vein of mice. The photograph shows metastatic tumors in the lung from each group (*top*) and the weight of whole lung tumors was measured from the two groups (*bottom*). (**F**) Metastasis in the lung from WT or ADAM9 KO TC1 cells injection intravenously appeared as dense tumor clusters (T) following hematoxylin and eosin staining (H&E). (**G**) Lung tumor tissues were processed for IHC staining of ADAM9. Images were scanned with Aperio software; digital resolution is 0.5 μm per pixel. Scale bar is 500 μm. Vessels were highlighted by red broken line (left, upper). The representative images of ADAM9 staining in tumor with or without vessels were showed (left, lower). The scale bar is 100 μm. The intensities of ADAM9, H score, were quantified by Aperio ImageScope software program (right). *P < 0.05, **P < 0.01, ***P < 0.001.

To further investigate the role of ADAM9 *in vivo*, we knocked out ADAM9 in TC1 mouse lung cancer cells using a CRISPR/Cas9 system. Expression of ANGPT2 and VEGFA was significantly reduced in two individual ADAM9 knockout (KO) TC1 clones compared to ADAM9 wild type (WT) cells (Fig. 4A). Next, we transplanted ADAM9 WT and KO TC1 cells into mice by subcutaneous injection and monitored the subsequent tumor growth. Mean tumor size was significantly smaller in the ADAM9 KO group than the WT group on day 22 (Fig. 4B). Expression of both ANGPT2 and VEGFA was significantly lower in ADAM9 KO tumors, as shown by western blotting (Fig. 4C). In addition, we stained for the endothelial marker CD31 to detect angiogenesis in tumor specimens and found lower expression of CD31 in the ADAM9 KO tumors (Fig. 4D). Finally, we transplanted ADAM9 WT and KO TC1 cells by intravenous injection into mice and observed lower lung tumor weight (Fig. 4E) and lung tumor burden (Fig. 4F) in the ADAM9 KO group than in the WT group. Notably, in metastatic lung tumors from TC1-WT group, we found higher expression of ADAM9 was significantly detected in well vascularized tumor nodules compared with tumor nodules without vessels by measuring H scores (Fig. 4G). These results suggest that ADAM9 knockout suppressed TC1 tumor progression, including tumor growth and metastasis, probably through reducing angiogenesis. Based on the preceding results, this is likely due to the influence of ADAM9 on ANGPT2 and VEGFA expression.

### Patients with simultaneous high ADAM9/VEGFA or ADAM9/ANGPT2 expression in primary lung adenocarcinoma have a poor prognosis

Higher angiogenesis is a characteristic of malignancy during tumorigenesis. To determine whether high levels of ADAM9-regulated ANGPT2 and VEGFA in lung adenocarcinoma patients are clinically relevant in terms of prognosis, we analyzed three lung cancer datasets (Asia population: GSE8894 and GSE11969; Western population: Shedden dataset) using a Cox proportional hazards regression model^19–21^.

**Figure 5:**
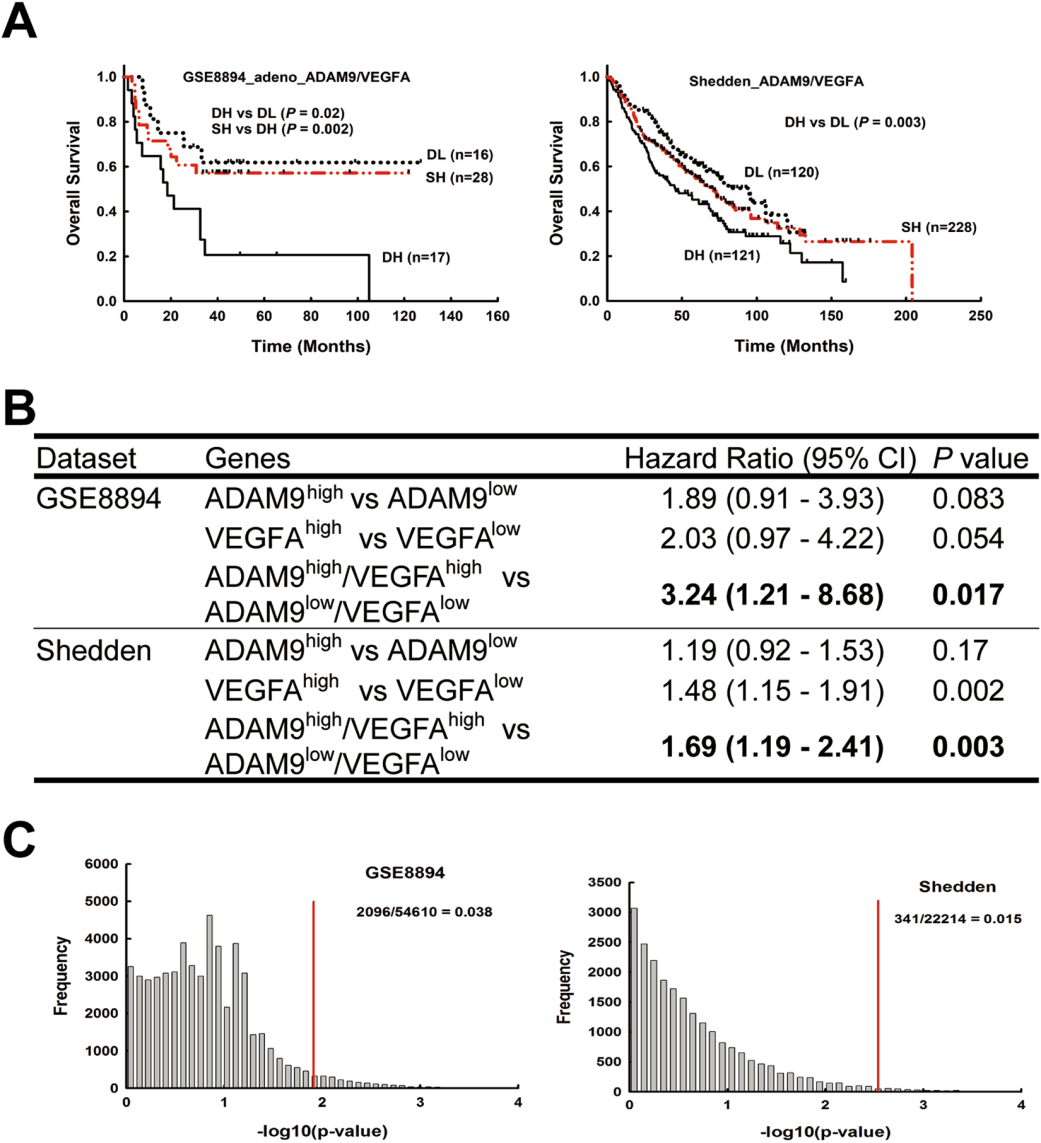
Simultaneous high expression of ADAM9 and VEGFA correlates with poor outcome in lung adenocarcinoma patients. (**A**) Kaplan-Meier survival analyses of lung adenocarcinoma patients in each indicated data set, with patients categorized by ADAM9 and VEGFA gene expression. DH, dual high; SH, single high; DL, dual low. High and low are above and below the median, respectively. (**B**) Cox proportional hazards regression analyses of overall survival in each dataset of lung adenocarcinoma patients, grouped by ADAM9 and VEGFA expression. (**C**) Ranking the *P*-value of ADAM9 plus VEGFA among all *P*-values of profile genes by combining a fixed predictor with the other randomly selected genes from the same dataset. Fixed predictor: ADAM9 in the GSE8894 dataset and VEGFA in the Shedden dataset. The *P*-value of ADAM9^high^/VEGFA^high^ versus ADAM9^low^/VEGFA^low^ is marked by the red line.

By combining ADAM9 and VEGFA as a predictor, patients with dual high expression of ADAM9 and VEGFA (ADAM9^high^/VEGFA^high^) had significantly less survival time compared to those with dual low expression of ADAM9 and VEGFA (ADAM9^low^/VEGFA^low^) in GSE8894 and Shedden datasets (Fig. 5A). (GSE11969 contains no results for VEGFA, so was not included in this analysis.) Moreover, Cox regression model analysis showed that patients with high levels of both ADAM9 and VEGFA have a significantly higher risk of mortality than patients with low levels of both genes (Fig. 5B). In the Shedden dataset, VEGFA expression alone was predictive of greater mortality (Fig. 5B). To verify that these results were not the product of random bias from a statistical error, a *P*-value profile was generated as previously described. The ranking of the *P*-values for the ADAM9-VEGFA combination was in the top 2–4% in the GSE8894 and Shedden datasets, indicating a low chance of random bias producing the results (Fig. 5C).

**Figure 6:**
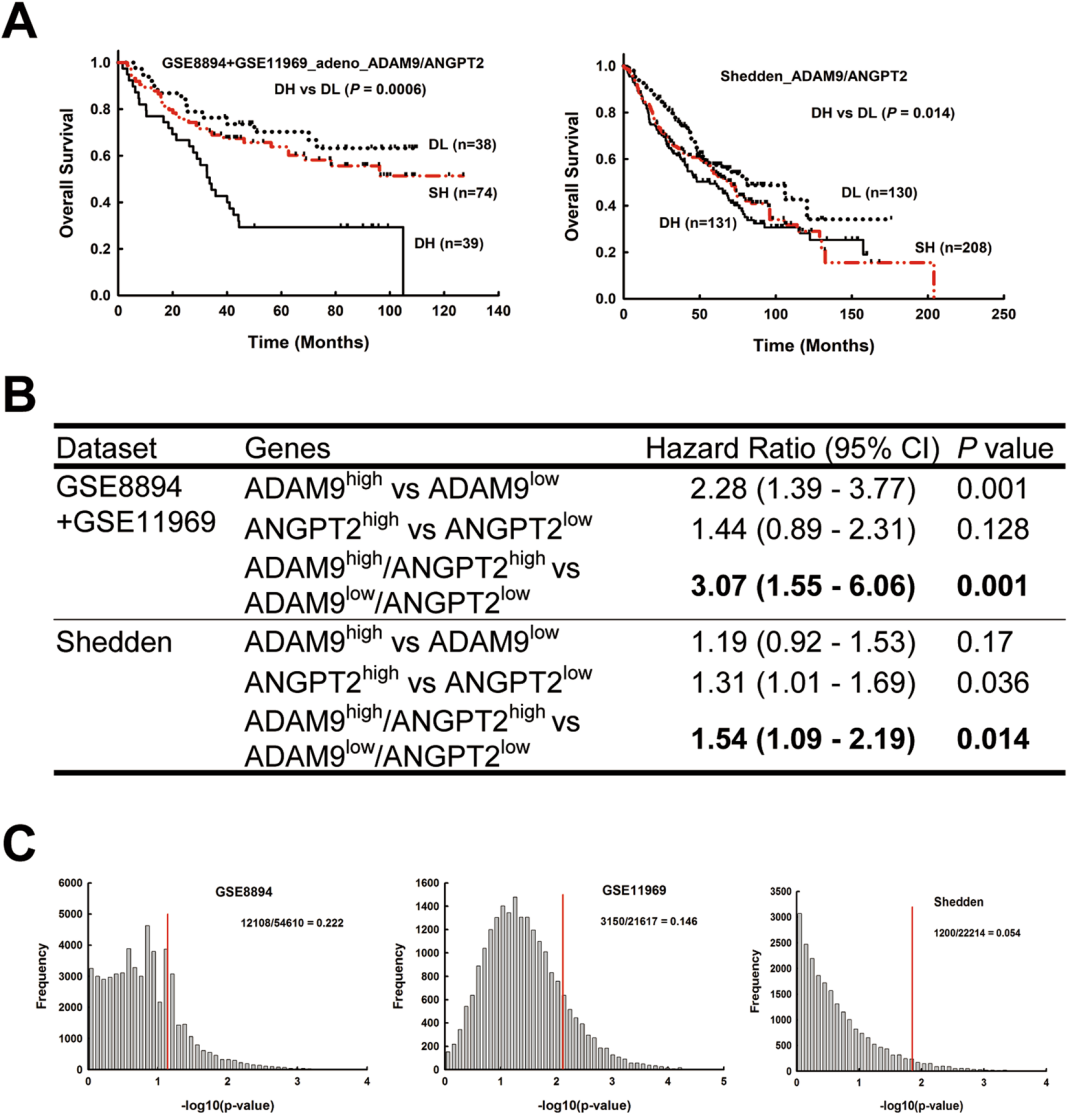
Simultaneous high expression of ADAM9 and ANGPT2 correlates with poor outcome in lung adenocarcinoma patients. (**A**) Kaplan-Meier survival analyses of lung adenocarcinoma patients in each indicated data set, with patients categorized by ADAM9 and ANGPT2 gene expression. Labels are defined as in Fig. 5. (**B**) Cox proportional hazards regression analyses of overall survival in each dataset of lung adenocarcinoma patients, grouped by ADAM9 and ANGPT2 expression. (**C**) Ranking the *P*-value of ADAM9 plus ANGPT2 among all *P*-values of profile genes by combining a fixed predictor with the other randomly selected genes from the same dataset. Fixed predictor: ADAM9 in the GSE8894 and GSE11969 datasets and ANGPT2 in the Shedden dataset. The *P*-value of ADAM9^high^/ANGPT2^high^ versus ADAM9^low^/ANGPT2^low^ is marked by the red line.

A similar trend was also found for ADAM9 and ANGPT2. ADAM9^high^/ANGPT2^high^ patients had lower survival rates compared to ADAM9^low^/ANGPT2^low^ patients in both Asian and Western population datasets (Fig. 6A). In addition, lung cancer patients with ADAM9^high^/ANGPT2^high^ had a significantly higher risk of mortality than did patients with ADAM9^low^/ANGPT2^low^ in both the Asia and Western datasets (hazard ratios [HR] of 3.07 and 1.54, respectively; Fig. 6B). This effect was driven primarily by ADAM9 expression in the Asian dataset (HR 2.28, *P* = 0.001) and by ANGPT2 in the Shedden dataset (HR 1.31, *P* = 0.036; Fig. 6B). The ranked *P*-value of dual high versus dual low expression of ADAM9/ANGPT2 was top 22%, 14%, and 6%, respectively, in the GSE8894, GSE11969, and Shedden datasets (Fig. 6C). Although the ranked *P*-value of ADAM9/ANGPT2 was slightly higher than that of the ADAM9/VEGFA combination, the value is still less than 25%, suggesting that the correlation of expression and mortality was not due to bias. Taken together, these results indicate that ADAM9^high^/VEGFA^high^ or ADAM9^high^/ANGPT2^high^ expression in lung tumors is associated with poor prognosis.

## Discussion

Metastasis of cancer cells to the brain requires several steps, including intravasation, overcoming anoikis, extravasation, vascular remodeling, and angiogenesis^3^. It is known that brain metastases occur at high frequency in lung cancer patients with advanced stage^22,23^, and that overexpression of ADAM9 is associated with brain metastases by increasing lung cancer cells’ adherence to the brain^13^. Our previous study has demonstrated that ADAM9 enhances cancer cells’ ability to survive in the blood and promotes metastasis by a plasminogen activator-based pathway in activating the anoikis-resistant gene *CDCP1* ^14^. Here, we showed that ADAM9 also contributes the functions of angiogenesis and vascular remodeling for cancer progression through regulation of VEGFA, ANGPT2, and PLAT via *in vitro* and *in vivo* experiments. Finally, we demonstrated that simultaneous high expression of ADAM9/VEGFA or ADAM9/ANGPT2 are linked to poor prognosis, which shows the clinical relevance of ADAM9-mediated vascular remodeling in lung cancer progression.

The BBB is composed of vascular endothelial cells and supporting components, such as the basal membrane, pericytes, and astrocytes, to protect the brain from toxins and pathogens^24^. Loss of the integrity of the BBB may increase the risk of cancer cells entering into the brain. Since tPA has the ability to disrupt the integrity of the BBB by a plasmin-dependent or plasmin-independent pathway^25,26^, the ability of ADAM9 to enhance the function of tPA may explain why high ADAM9 expression in lung cancer cells is associated with brain metastasis. In addition, ANGPT2 is associated with endothelial cell apoptosis and BBB breakdown^8,27^. In this study, we demonstrated that ADAM9-mediated effects on tPA and ANGPT2 influenced the integrity of brain endothelial cells, consistent with previous findings^8,26^.

Although ADAM9 has been reported to be involved in pathological retinal neovascularization by shedding membrane proteins from endothelial cells^16^, whether ADAM9 in cancer cells also influences new vessel formation has yet to be determined. In our study, a higher concentration of VEGFA was detected in ADAM9-abundant lung cancer cells compared to ADAM9 knockdown cells and resulted in higher angiogenesis in endothelial tube formation assays. Notably, we found the concentration of VEGFA in conditioned medium from the Bm7 cells to be almost 1000-fold lower than that typically used for tube formation *in vitro* (50 ng/mL)^28^, suggesting that VEGFA may not be the only pro-angiogenic factor affected by ADAM9. Future studies should seek to identify these other angiogenic factors.

The metalloprotease domain of ADAM9 can cleave the heparin-binding epidermal growth factor like growth factor (HB-EGF) precursor to yield soluble HB-EGF^29^, thereby activating epidermal growth factor receptor (EGFR) signal transduction to promote tumor growth and angiogenesis^30^. We also demonstrated ADAM9 knockdown reduced EGFR signaling activation and increased the sensitivity to EGFR inhibitor treatment^31^. Moreover, activation of EGFR signaling pathway on tumor cells is associated with angiogenesis by regulating pro-angiogenic factors^32,33^. Thus, these reports suggest that ADAM9 associated angiogenesis is likely through EGFR signaling. However, we demonstrated that ADAM9 overexpression of WT or MT can increase VEGF and ANGPT2 expression regardless the protease function, suggesting other mechanism was involved in regulating VEGF or ANGPT2 expression by ADAM9. Therefore, the detailed mechanisms for downregulation of VEGF and ANGPT2 are need to be further clarified.

Increased vessel density and elevated expression of VEGFA are significantly related to a poor survival in lung cancer^34,35^. Moreover, the relationship of high expression of ADAM9 with poor clinical outcomes has been investigated in various other human cancers^36–38^. ADAM9 mediates cellular responses in stressful environments such as cell crowding, serum starvation, hypoxia, and hydrogen peroxide^39–41^ to regulate processes which are correlated to tumorigenesis and metastasis. Recently, ADAM9 expression is tightly regulated by different microRNAs to modulate the process of tumorigenesis and metastasis in different cancer types^42–45^. Our results showed ADAM9 silence in lung cancer cells significantly reduce the VEGFA and ANGPT2 expression. Notably, smaller tumor growth and lung metastasis in ADAM9 KO TC1 groups compared to control groups were observed in mice bearing lung tumors. From the ADAM9 IHC staining, we demonstrated high levels of ADAM9 are associated with blood vessel formation, suggesting ADAM9 promotes angiogenesis for tumor progression. The observation in animal models is consistent with the clinical dataset analyses that poor outcomes in lung cancer patients with simultaneous high expression of ADAM9 and VEGFA or of ADAM9 and ANGPT2.

Together, these findings provide evidence to explain how ADAM9 modulates metastases through controlling vascular remodeling and angiogenesis. By linking the function of ADAM9 to clinically relevant outcomes in lung cancer patients, we have demonstrated that ADAM9 could be useful as both a prognostic biomarker and as a molecular target for reducing cancer progression. However, potency of metalloproteinase inhibitors for inhibition of ADAM9 including marimastat, batimastat (BB94), GM6001, TAPI-2, GW280264X were acted as a broad-spectrum matrix metalloproteinase inhibitor, not specific for ADAM9^17^. In addition, RAV-18, an ADAM9-specific blocking antibody, showed *in vivo* antitumor activity in a gastric cancer xenograft model^40^. Although our results demonstrate ADAM9 proteins regulate VEGFA and ANGPT2 expression regardless protease activity, shedding function of ADAM9 is still important for pro-angiogenic factors generation to promote tumor growth. We believe that ADAM9 inhibitions in protein expression and protease activity are potential therapeutic approaches for treatment of lung cancer.

## Methods

### Cell culture and reagents

The Bm7 and Brmx2 human lung cancer cell line was established as previously described^14^. The mouse lung cancer cell line TC-1 was kindly provided by Dr. T.C. Wu^46^. A549 cell line was obtained from Bioresource Collection and Research Center (Hsinchu, Taiwan). Human umbilical vein endothelial cells (HUVECs) was kindly provided by Dr. Jeng-Jiann Chiu in National Health Research Institute, Taiwan. Human brain microvascular endothelial cells (HBMECs) and primary human astrocytes are purchased from ScienCell Research Laboratory (Carlsbad, CA, USA). Experiments were performed within 6 months of receipt from the cell bank to ensure cells’ ability. All cells used in this study were confirmed to be mycoplasma-free. The antibodies used in the study were against ADAM9 (MAB939, R&D Systems, Minneapolis, MN, USA), mouse ADAM9 (AF949, R&D Systems), ANGPT2 (AF623, R&D Systems), VEGFA (ABS82, Millipore, Billerica, MA, USA), CD31 (ab28364, Abcam), and elongation factor 1 α (EF1α, #05–235, Millipore). The activity of PLAT in different dilutions of the conditioned medium was detected with the AssaySense Human PLAT Immuno-Chromogenic Activity Assay Kit (Assaypro, St. Charles, MO) in 2-day cultured cells.

### Gene knockdown or knockout

All methods were carried out in accordance with relevant guidelines and regulations in China Medical University, Taiwan. Knockdown of ADAM9 was performed as previously described^14^ using three lentiviral vectors (clone C: TRCN0000046980; clone E: TRCN0000046982; clone H: TRCN0000290528). The reagents were obtained from the National RNAi Core Facility located at the Institute of Molecular Biology, Genomic Research Center, Academia Sinica in Taipei. A CRISPR RNA-guided Cas9 nuclease gene targeting system was used to knock out the ADAM9 gene in mouse TC1 and human Brmx2 lung cancer cells with a ToolGen kit (ToolGen Inc.) according to the manufacturer’s protocol. We manually searched for CRISPR target sequences consisting of G(N)_19_NGG at the web site crispr.mit.edu. The expressed guide oligos (for targeting to mice genomic DNA: 5′-GGAGGGAGTGCAGAATTCCGCGG-3′; human genomic DNA: 5′-CGGGGACCCTTCGTGTCCGGTGG-3′) were used for constructing targeting sites with which to knock out ADAM9. The efficiency of ADAM9 knockdown or knockout was tested by western blot analysis.

### Plasmid construction and gene expression

Human mature ADAM9 cDNA (consisting of nucleotides 616–2538) with HA-tag in N-terminal was amplified by polymerase chain reaction from Bm7 lung cancer cell line and cloned into the pLNCX vector. The activity mutant ADAM9 in which the catalytic site consensus sequence HEXXH was mutated to HAXXH (E348A)^17^ was generated using a site-directed mutagenesis kit (Agilent Technologies, Santa Clara, CA, USA) according to the manufacturer’s protocol. Both two plasmids were confirmed by DNA sequencing. Brmx2 cells were transient transfected with an empty vector (E) or the vector pLNCX carrying human ADAM9 (WT), activity mutant ADAM9 (MT) using Lipofectamine 2000 reagent (Invitrogen, Carlsbad, CA, USA).

### Transcriptome microarray analysis

To analyze the RNA profile, Illumina Human-6 v3 BeadChips (Illumina, San Diego, CA) were used as previously described^14^. We detected the expression levels with the Illumina BeadArray Reader and then analyzed them in the National Taiwan University Microarray Analysis Platform & System (NTUMAPs) with commercial software Partek^® ^^47^. Ingenuity Pathway Analysis (Ingenuity Systems, Inc.) was carried out to explore gene-gene interaction networks, functions, and canonical pathways.

### Quantification of VEGFA protein by ELISA

VEGFA protein concentration was measured by a Human VEGF DuoSet kit (DY293B, R&D Systems, Minneapolis, MN, USA). All experiments were performed in triplicate according to the manufacturer’s instructions.

### Endothelial tube assay

The detailed procedures have been previously described for *in vitro* angiogenesis^48^. Briefly, HUVECs pretreated with conditioned media from indicated cells were seeded onto Matrigel pre-coated plates. After 4-h incubation, capillary-like structures formed, and images were captured with a Nikon camera at 100X magnification. The number of tubes formed or total tube length in each well was measured by the ImageJ program (National Institutes of Health, USA).

### Immunofluorescence staining

Human brain microvascular endothelial cells (HBMECs) were seeded onto fibronectin pre-coated 12-well μ-slides (ibidi GmbH, Martinsried, Germany) with a cell density of 8 × 104/mL for overnight culturing. HBMECs were then treated with concentrated conditioned medium from control and knockdown cells for 24 h. To neutralize ANGPT2, the anti-ANGPT2 antibody (10 μg/mL; AF623, R&D) was added to the concentrated conditioned medium for 1 h of incubation, and then this medium was used to treat HBMECs. After culturing, cells were fixed with 4% paraformaldehyde in PBS for 20 min at room temperature and permeabilized with 0.1% Triton X-100 in PBS (PBS-T) at 4 °C for 10 min. After blocking with 4% BSA in PBS at 37 °C for 30 min, cells were stained with anti-human VE-cadherin antibody (#2158, Cell Signaling) and rhodamine conjugated anti-rabbit IgG (Jackson ImmunoResearch Laboratories, West Grove, PA). DAPI was used for nucleus staining. Slides were mounted and viewed under a fluorescence microscope (Zeiss Axio Observer Z1) and analyzed using the AxioVision software (Zeiss). The length of VE-cadherin reflected by fluorescence was calculated by the ImageJ program and normalized to the cell number.

### Blood-brain barrier permeability assessment

This assay was performed as previously described^25^. Briefly, HBMECs and primary human astrocytes (ScienCell, Carlsbad, CA, USA) were prepared as an *in vitro* trans-blood-brain barrier (BBB) model on 0.4 μm pore-size filters. The inserts were transferred into new wells containing M199 medium, and conditioned medium from cancer cells was added into the upper well 1 h before addition of FITC-conjugated BSA (1 μg/μL, Sigma) for a 45 min incubation. In the ANGPT2 antibody-treated group, 10 μg/mL of anti-ANGPT2 (AF623, R&D Systems) was added to the conditioned medium for a 1 h incubation before adding to the HBMECs. The fluorescence in the lower wells was measured to monitor the integrity of the BBB by the multi-mode microplate reader (BioTek Instruments, Winooski, VT, USA).

### Animal studies and tumor analysis

All methods were carried out in accordance with relevant guidelines and regulations in China Medical University, Taiwan. All animal experiments were approved by the Institutional Animal Care and Use Committee of China Medical University and Hospital, Taiwan. The female B6 mice were purchased from National Laboratory Animal Center (Taipei, Taiwan). For the mouse lung cancer models, 1 × 10^6^ wild-type (TC1-WT) or ADAM9 knockout (TC1-ADAM9 KO) TC1 cells were injected subcutaneously into the flanks of C57BL/6 mice at 6 weeks of age. The size of subcutaneous tumors was calculated by the formula *V* (mm^3^) = *a* × *b* ^2^/2, where *a* is the largest dimension and *b* is the perpendicular diameter^49^. For the metastatic model, TC1-WT or TC1-ADAM9 KO cells (1 × 10^6^) were intravenously injected via the tail vein of the mouse at 6 weeks of age. All mice were sacrificed 28 days after inoculation of the tumor cells. Tumors were dissected from syngeneic tumor mice and used for western blot analysis to detect protein expression of ADAM9, ANGPT2, and VEGFA. Tumor specimens were fixed in 4% neutral-buffered formaldehyde and embedded in paraffin. Immunohistochemical (IHC) staining of CD31 or ADAM9 was performed using CD31 antibody (ab28364, Abcam, Cambridge Science Park, Cambridge, UK) or ADAM9 antibody (AF949, R&D systems), horseradish peroxidase-conjugated avidin/biotin complex from the Vectastain Elite ABC Kit (Vector Laboratories Inc., Burlingame, CA, USA), and AEC chromogen (Vector Laboratories). After hematoxylin counter-staining, sections were mounted and examined under a microscope (Leica DM500, Heerbrugg, Switzerland). To quantify CD31 stained areas, slides were photographed randomly by LAS version 4.4.0 (Leica). After subtracting the non-specific signal, the selective reliable red signal areas in each view were measured by the ImageJ program. For automated scoring the expression of ADAM9, the slide was scanned using an Aperio Scanscope Scanner (Aperio Vista, CA, USA) and viewed through Aperio ImageScope software program (Aperio Technologies).

### Clinical dataset analyses

We analyzed the gene expression of lung adenocarcinoma patients from three available datasets as previously described^50^: the GSE8894^19^ and GSE11969^51^ datasets from the Gene Expression Omnibus^52^, and the dataset from the Shedden *et al*. study^53^. All the datasets were normalized to remove the potential systematic biases. Overall survival was analyzed for the genes of interest by dividing patients into two groups according to gene expression level (above or below the median).

### Statistical analysis

To compare the analyzed parameters between control and experimental groups, we used 2-sided Student’s t-tests or two-way ANOVA for continuous variables. The log-rank test was used to determine survival differences, and a Cox hazard regression model was used to quantitate the risk of the patients with lung adenocarcinoma according to gene expression level. For all tests, statistical significance was defined as *P* < 0.05.

## Electronic supplementary material

Supplementary Figure 1
