# Supplementary material for: ADAM9 promotes lung cancer progression through vascular remodeling by VEGFA, ANGPT2, and PLAT
Source: Sci Rep. 2017 Nov 8;7:15108. doi: 10.1038/s41598-017-15159-1 (PMC5678093; doi:10.1038/s41598-017-15159-1)
Supplement: Supplementary file 1 — Supplementary Figure 1 [file 41598_2017_15159_MOESM1_ESM.pdf]

ADAM9 promotes lung cancer progression through vascular remodeling by VEGFA, ANGPT2, and PLAT

Chen-Yuan Lin, Chia-Fong Cho, Shih-Ting Bai, Jing-Pei Liu, Ting-Ting Kuo, Li-Ju Wang, Yu-Sen Lin, Ching Chan Lin, Liang-Chuan Lai, Tzu-Pin Lu, Chih-Ying Hsieh, Chin-Nan Chu, Da-Chuan Cheng, Yuh-Pyng Sher

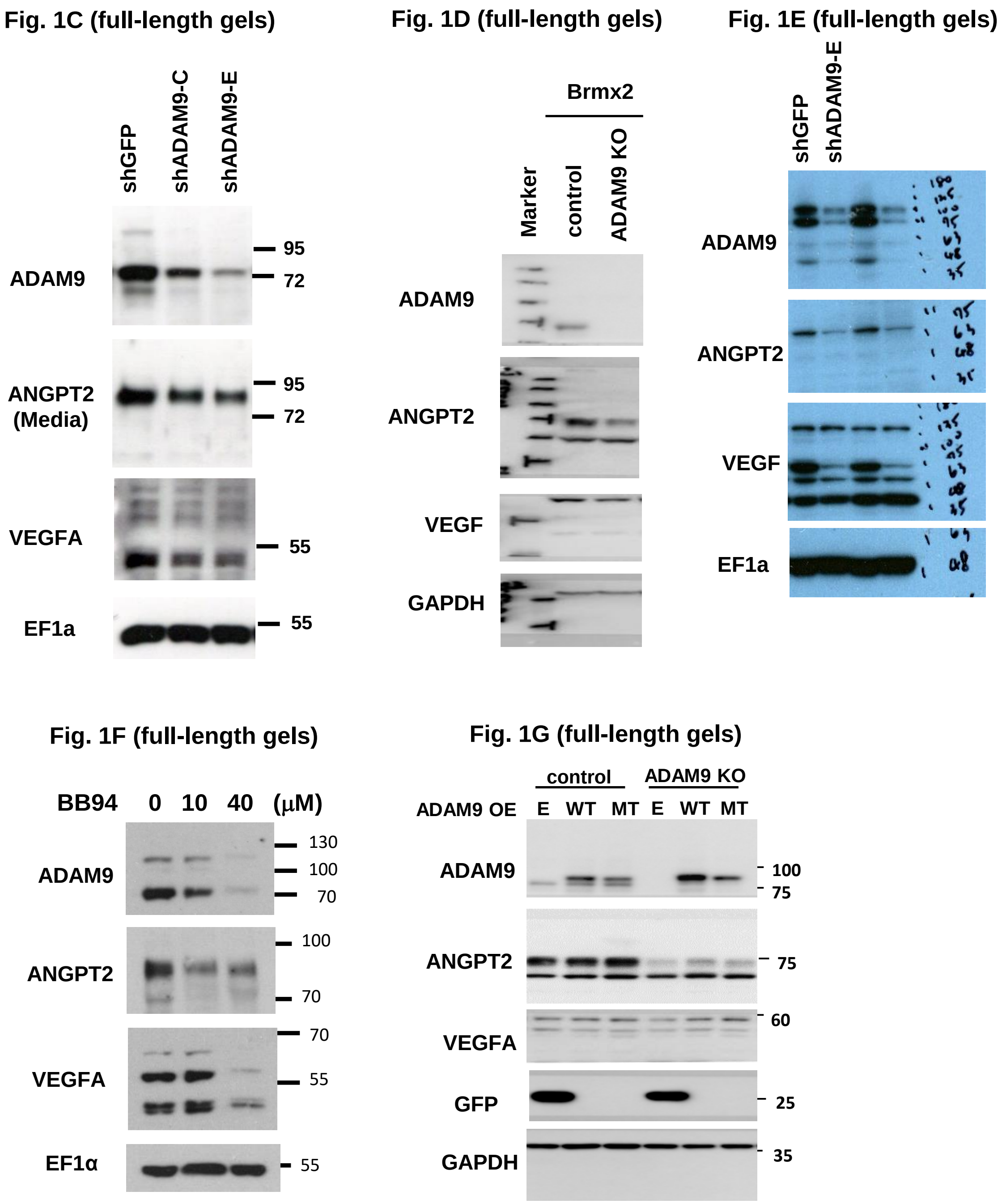

Supplementary Figure 1. Full-length blots of Figure 1.
